# Press-Fit Placement of a Rectangular Block Implant in the Resorbed Alveolar Ridge: Surgical and Biomechanical Considerations

**DOI:** 10.3390/bioengineering11060532

**Published:** 2024-05-23

**Authors:** Efthimios Gazelakis, Roy B. Judge, Joseph E. A. Palamara, Shiva Subramanian, Mohsin Nazir

**Affiliations:** 1 Melbourne Dental School, University of Melbourne, Parkville, VIC 3052, Australia; nazirm@student.unimelb.edu.au; 2 Department of Prosthodontics, Melbourne Dental School, University of Melbourne, Parkville, VIC 3052, Australia; roybj@unimelb.edu.au; 3 Restorative Dentistry, Melbourne Dental School, University of Melbourne, Parkville, VIC 3052, Australia; palamara@unimelb.edu.au; 4 Oral and Maxillofacial Surgery, Melbourne Dental School, University of Melbourne, Parkville, VIC 3052, Australia; shivass@unimelb.edu.au

**Keywords:** rectangular block implant, rectangular osteotomy, primary retention, placement load, osseo-integration, stress concentration

## Abstract

Rectangular Block Implant (RBIs) were manufactured, using computer-aided-design lathe turning, surface roughened with grit blasting and gamma irradiated. Implants were surgically placed into the resorbed edentulous mandibular ridges of both greyhound dogs (ex vivo and in vivo) and humans; the pooled total was 17 placements. The aim was to achieve mechanical stability and full implant submergence without damage to the mandibular canal and without bone fracture: fulfilment of all of these criteria was deemed to be a successful surgical outcome. Rectangular osteotomy sites were prepared with piezo surgical instrumentation. Sixteen implants were fully submerged and achieved good primary stability without bone fracture and without evidence of impingement of the mandibular canal. One implant placement was deemed a failure due to bone fracture: the event of a random successful outcome was rejected (*p* < 0.01 confidence, binomial analysis). Technique of placement yielded excellent mechanical retention: key biomechanical factors that emerged in this process included under preparation of the osteotomy site with the use of specifically designed trial-fit gauges, the viscoelastic property of the peri-implant bone, the flat faces and cornered edges of the block surfaces which enhance stress distribution and mechanical retention, respectively. It was concluded that the surgical protocol for the RBI placement in the resorbed alveolus is a predictable clinical procedure tailored to its specific, unique biomechanical profile.

## 1. Introduction

The surgical placement of dental implants has developed extensively over the past decades to evolve into a predictable clinical procedure [1]. Initially, full submergence of fixtures was mandated, predicated on intra-osseous cortical engagement. Later requirements simply needed 2 mm of buccal and lingual crestal ridge width [2]. Many clinicians have dispensed with the above and claimed successful clinical outcomes with simultaneous grafting on placement of significant ridge deficiencies and implant exposures on initial placement [3]. Although this final step remains equivocal, the critical factor claimed by all is mechanical (primary) stability [4]. The micromotions resulting from a failure to achieve mechanical primary stability have been shown to result in a lack of osseo-integration and the formation of a fibrous capsule [5].

Surgical intra-operative complications, in turn, may play a significant role in compromised primary stability. Prime examples of this are bony fractures at the time of implant placement [6,7] and over-preparation of the osteotomy site [8]. Specifically with regard to mitigating over-preparation, Varcellotti [9] has summarised that the piezo-surgical micro-metric cutting action is extremely precise, eliminating the chatter of conventional drilling, due to the physical phenomenon of cavitation used by the ultrasonic action (frequency = 29 kHz; amplitude= 60–200 μm). This is a predictable and reliable method of bone preparation where precise linear cuts are possible.

This implant surgical landscape is dominated by “vertically oriented” cylindrical implants that geometrically match the tooth root structure [10]. Historical examples have demonstrated, however, that implants can be of varied geometries [11,12], and this is even further magnified in post-neoplastic reconstructive procedures [13]. Indeed, the entire biomechanical concept of root-crown length ratios has been challenged [14].

Recently, a rectangular block implant (RBI) has been developed for situations where the alveolar ridge saddle is resorbed. It is “horizontally oriented” and designed to maximise force-load distribution, mechanical stability, surface area bone contact, and preservation of vital anatomical structures. This mandates, however, the surgical creation of a rectangular osteotomy site and a press-fit insertion technique [15].

Although the use of press-fit implants is not a new phenomenon [16], there is a paucity of studies that clarify the biomechanical characteristic of this technique, which is, in essence, an implant–bone mechanical interference fit. Surgical orthopaedic literature has highlighted that the press-fit implant placement technique is not without limitations [17]. This is based on an unavoidable yet undefined degree of permanent bone deformation [18]. Further undefined elements include the influence of bone quality, the degree of implant roughness [19], the necessary degree of interference mismatch [20] and the remaining degree of implant–bone interface “gaps” [21].

The bone tissue has been typically modelled as an isotropic linear elastic material, and press-fit placement of implants has been modelled as a complex interplay between elastic and plastic deformation of the surrounding bone [22]. Key bone parameters are Young’s modulus (E) = 15 GPa, Poisson’s ratio (ν) = 0.3, and density (ρ) = 731 mg/cm^3^ [20], while the titanium structure is typically almost one order of magnitude greater in stiffness (E = 115 GPa). Pertinent biomechanical issues here are the viscoelasticity and nature of the bone itself, the degree of dimensional under-preparation of the osteotomy, and the compressive forces generated [20]. These factors typically relate to cortical bone, which dominates in the resorbed ridge. The intra-osseous implantation of the RBI is a balanced interplay between these biomechanical issues; failure to achieve this balance results in possible bone fracture/damage [23], loss of primary retention, or extra-osseous (“exposed”) implant surfaces.

The aim of this article is to describe the surgical placement of the RBI and, in particular, clarify the biomechanical aspects in response to the placement loads that underpin the success of this surgical procedure, based on three surgical outcomes: complete intra-osseous coverage of the RBI, the absence of bony fractures and achievement of initial mechanical stability.

## 2. Materials and Methods

### 2.1. Manufacture

**Figure 1:**
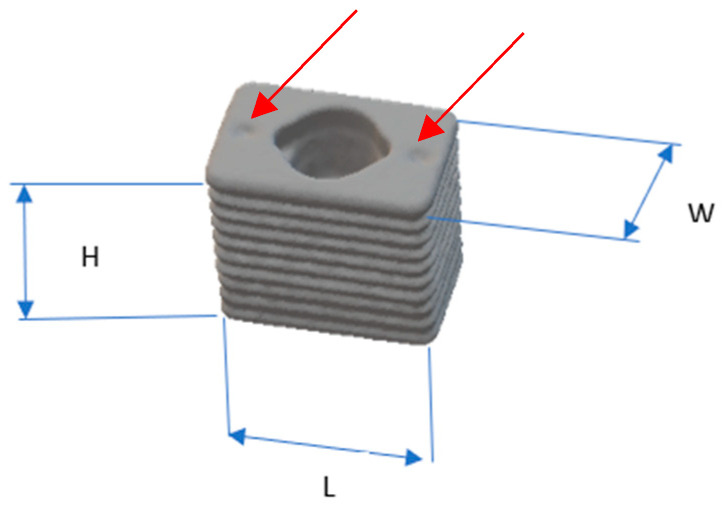
RBI dimensions: 6 mm in “horizontal” mesiodistal length (L), 5.25 mm in “vertical” crestal-apical height (H), and 4 mm in “horizontal” bucco-lingual width (W). Mesial and distal crestal surface depressions (red arrows) facilitate the press action fit with the use of centre punch action [24].

RBIs were manufactured from Gr IV titanium rods using computer-aided design (CAD) and then computer-aided manufacture (CAM) [combined: CAD–CAM] lathe turning. Surface treatment was completed using Al_2_O_3_ grit (animal placement RBIs) and TiO_2_ grit (human placement RBIs) blasting (Physics Department, Royal Melbourne Institute of Technology) followed by hot bath acid etching (Melbourne Dental School, University of Melbourne), whereupon implants were cleansed, dust free packaged and then sent for gamma irradiation (Steritech, Melbourne). The design of the RBI (Figure 1) has been previously presented [24,25].

**Figure 2:**
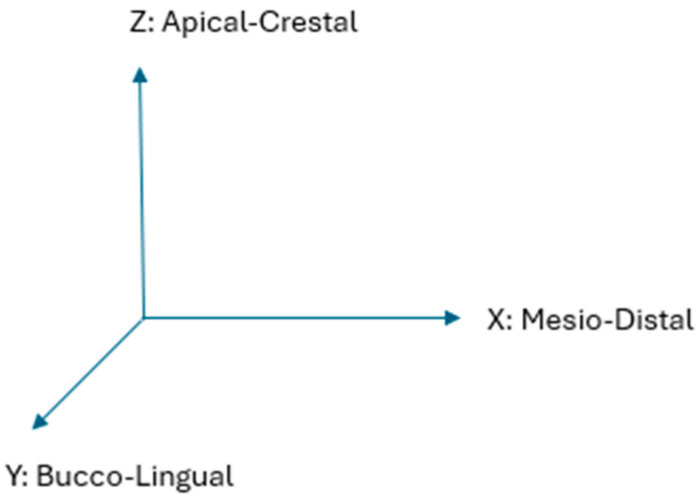
Corresponding anatomical axes X, Y, Z. Where the “horizontal” axes are X and Y, while the “vertical” axis is Z.

Corresponding with the above dimensions of the length, width and height, the implant and surrounding bone aspects have been assigned *X*, *Y* and *Z*-axes, respectively. These further correspond anatomically to “horizontal” mesiodistal, “horizontal” buccolingual and “vertical” crestal-apical ridge aspects, respectively (Figure 2).

### 2.2. Experimental Subjects

A total of 17 placements were performed. The experimental phases consisted of two stages: firstly, placement in the greyhound dog animal model (both ex vivo and in vivo) and secondly, in two human patients.

#### 2.2.1. Ex Vivo Animal Placements (×3)

These were performed on the inferior border of the mandible of animals that had been recently euthanised through use in other unrelated medical research within the University of Melbourne and which had not compromised the head of the animals. The inferior border was chosen as there were no edentulous saddles in these animals, and there is a close proximity from this border to the mandibular canal (housing the inferior alveolar neuro-vascular bundle [IAN]). All ex vivo placements involved de-fleshing the inferior mandibular border.

#### 2.2.2. In Vivo Animal Placements (×12)

These were performed at the School of Veterinary Science (University of Melbourne) Animal Clinic (Ethics ID: 1112344.1). All animal and animal tissue handling (both ex vivo and in vivo) was executed in accordance with the principles of Enhancing the Quality and Transparency Of health Research (EQUATOR) [26] as applied to animal experimentation: Animal Research Reporting In Vivo Experiments (ARRIVE).

Three mature greyhound dogs were chosen from the Veterinary School, University of Melbourne facility’s dog colony: two males and one female. Four months prior to the commencement of the implant placements, the necessary dentoalveolar arch room was achieved in each animal through left and right mandibular mid-arch molar (carnassial) extractions, leaving sufficient teeth for ongoing masticatory function.

Animals were anaesthetised with Propofol induction (approximately 10–15 mL via a cephalic vein catheter) and maintained with oxygen–Isofluorane endotracheal intubation (1.5–2.0%).

#### 2.2.3. Human Placements (×2)

These were performed chairside, under infiltration and block local anaesthesia (2% Lignocaine with 1:80,000 Adrenalin), after evaluation with cone beam computerised tomography (CBCT) evaluation [Human Ethics ID:1955302.1, University of Melbourne]. Consent was based on the guidelines of a Plain Language Statement discussed pre-operatively [27]. Ridge sites chosen were based on the prerequisite of clinically evident alveolar resorption greater than 5 years post extraction. Exclusion criteria included all local, systemic and pharmacological factors which would compromise bone quality, including periodontal, neoplastic and metabolic diseases.

### 2.3. Implants

The critical aspects of this design relevant to the current presentation are the surface depressions (Figure 1) on the crestal face of the RBIs, which, together with the central shaft geometry, allowed 3 positions through which to tap into the RBIs into their final positions with centre-punch and mallet action.

### 2.4. Site Assessment

In animal subjects, this was only possible through clinical evaluation at the time of placement. In human subjects, this was performed through CBCT, which allowed visualisation of the osteotomy site and fabrication of a surgical guide (CAD-CAM) to mark the ridge outline of the proposed site.

### 2.5. Flap Design

**Figure 3:**
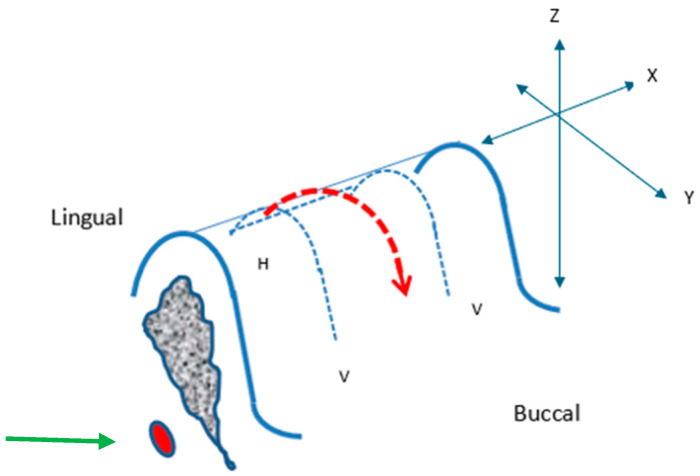
Surgical Protocol: Incision Design. Vertical, V; Horizontal, H; incisions (blue dashed lines). Raised soft tissue flap overlying the crest (red arrow dashed outline). X, Y, and Z-axes and the position of the inferior alveolar nerve (IAN) (green arrow) are depicted.

This was only relevant for in vivo greyhound and human placements. An incision was planned on the buccal aspect at both ends of the edentulous segment (where the placements were to take place), perpendicular to the crest of the ridge (“vertical, V” relieving) and a connecting mesiodistal (“horizontal, H”) incision was on the crest, lingually oriented, connecting the buccal incisions (Figure 3).

A periosteal elevator was used to raise the soft tissue flap overlying the crest (red dashed outline), starting from the lingual crestal position extending mesiodistally and then buccally, raising the entire segments of mucoperiosteum, and exposing the underlying bony surface of the segment.

### 2.6. Rectangular Osteotomy Construction

This was achieved through the use of specialised commercial piezotomes on a piezo-electric ultrasonic surgical unit (Mectron Piezosurgery, Mectron Corporation, Australia). Initial preparation was through the use of a saw action insert to mark the mesiodistal borders, the mid-crestal length and the depth of the osteotomy site as micro-metric cuts.

The use of the laser depth marks on the piezotome saw-ended insert was helpful in marking the mesial and distal extremities of the intended osteotomy, as well as its depth.

This was followed by rectangular prismatic-shaped inserts to initially widen the trough and then the spade-shaped insert to yield the full width of a rectangular trough. Once having established mesiodistal and buccolingual margins to the required depth of approximately 5 mm, the remaining depth “block of bone” was elevated out by lateral pressure to the full depth of the initial cuts to fracture the underlying trabecular attachment. The result was a uniform rectangular trough.

The buccolingual width advancement was performed in steps using the spade-shaped piezotomes.

Initial cuts were commenced on the lateral border of the mandible, and the block was used to gauge the size of these cuts.

**Figure 4:**
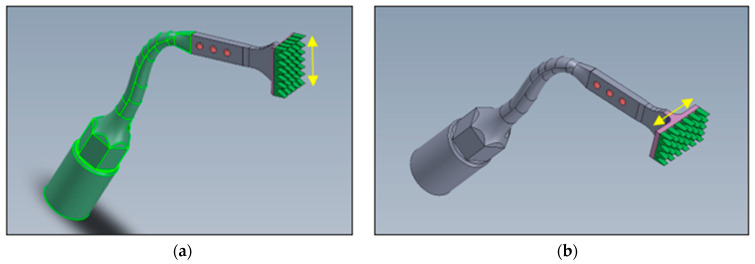
Bespoke Rectangular Piezotomes. Two such designs were manufactured: (**a**) where the action is in a buccolingual direction (Y-axis) and (**b**) the other in a mesiodistal direction (X-axis). The aim of these tools was to facilitate the final flat rectangular osteotomy at the full depth (5.25 mm). Yellow arrows represent relative 5 mm lengths.

The human placement osteotomies were completed with bespoke piezo tools (Patent Pending: Australian Patent Office: 2022902930) that had flat rectangular cutting platforms with rows of uniquely designed cutting teeth (0.5 mm high) [Figure 4].

### 2.7. Implant Placement and the Trial-Fit Gauge

**Figure 5:**
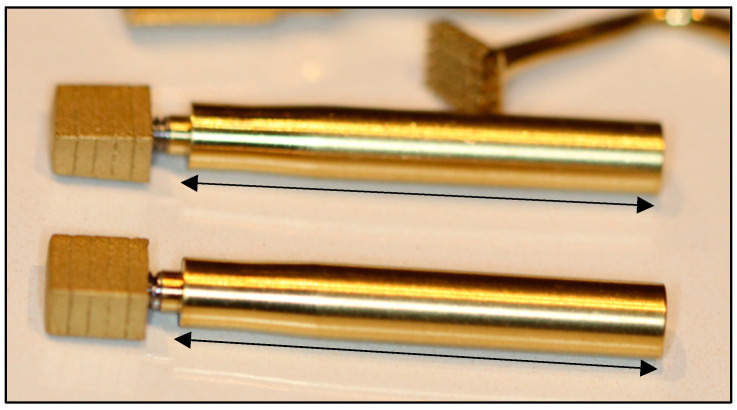
Trial-fit Gauges with 20% depth markings and stems attached. Black arrows represent relative 3.0 cm lengths.

A trial-fit gauge was needed for the repeated testing of the developing site, such that the fit approximated complete seating without tapping. The trial-fit gauge (Figure 5) was a duplicated implant that was not surface treated (i.e., it was machine finish only) and hence had a lower coefficient of friction upon insertion-removal.

It was designed such that its dimensions were to be 0.25 mm less than the intended implants in both mesial and distal surfaces as well as both buccal and lingual surfaces. Its overall dimensions were thus 0.5 mm shorter in the mesiodistal aspect and 0.5 mm narrower in the buccolingual aspect.

Premature attempts to tap/press-fit the implant in the under-sized rectangular defect threatened buccolingual bone fracture. It was essential to develop the site to the point where final tapping would yield complete parallel-planar seating, complete immobility and without bony fracture.

The trial-fit gauge thus spared the repeated use of the final implant, whose surface may have become contaminated with debris and other micro-organisms through the repeated handling of trial-fits and removals.

After trial-fit gauge removal, the block was introduced via its attached stem and passively inserted before tapping to determine the degree of fit. An under-prepared site would cause early binding, which, upon tapping, could result in the risk of fracture. The level of this passive fit at which point tapping could be commenced was determined at approximately 75% of the overall height.

A 3 cm stem attached to the internal thread of the trial-fit gauge was used as a removal tool designed as a rescue method, which would then allow rectification of the osteotomy site to facilitate complete insertion. The intended final outcome was one where the block would be firmly seated into position with mesiodistal tapping actions without bone fracture and where the seated RBI crestal surface would be flush with the “crestal” bone level.

### 2.8. Primary Stability

This was assessed through firm medio-distal and buccolingual finger (approximately 300 g force) pressure on the tip of the placement stem at its furthest point from the RBI while re-attached to the RBI body after mallet insertion. Any movement was deemed to be a failure of mechanical stability. With animal placements this was also assessed with torque testing at the time of placement, to 35 Ncm.

### 2.9. Radiography

Animals were euthanised (20 mL of sodium pentobarbitone [325 mg/mL]) 3 months post placement. CBCT radiographs were taken after each of the animal placements. Bucco-lingual sectional images were generated to visualise the apical aspect of the placements and the bucco-lingual bony walls. Human radiographs were taken pre-operatively with CBCT and post-operatively with routine dental in-office radiology.

### 2.10. Surgical Protocol Summary

**Figure 6:**
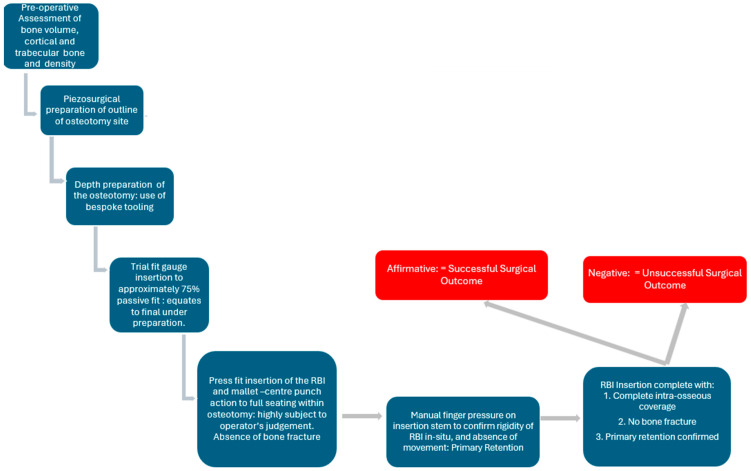
The developed RBI surgical protocol in the assessment of final biomechanical successful press-fit placement.

The above principles were applied as an overall surgical protocol (Figure 6).

## 3. Results

### 3.1. Ex Vivo Animal Placements

**Figure 7:**
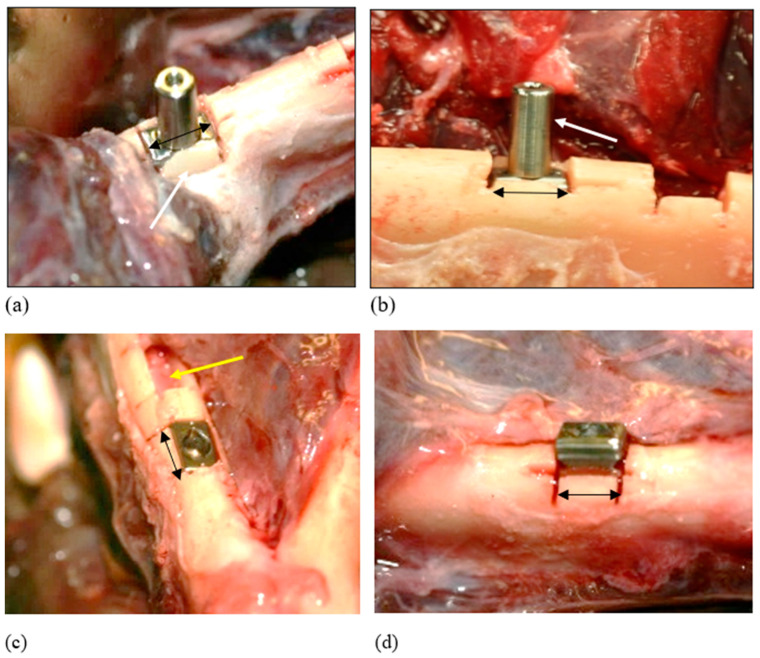
Successful Insertion: Ex vivo. These cases highlighted the elasticity of the bone. (**a**) The cortical plate here overlapped the crestal aspect of the inserted block implant. Instead of fracturing on tapping into place, the bone expanded and accommodated the implant (white arrow). (**b**) There was excellent primary retention, as felt by attempting to move the implant through its attached abutment (white arrow). (**c**) Complete seating of the RBI and evidence of the undamaged underlying inferior alveolar nerve (yellow arrow). (**d**) Incomplete passive seating: Less than 75%. This level of initial passive seating was deemed inadequate, resulting in fracture. Black arrows represent relative 6 mm lengths.

Three placements were attempted: two reached successful seating (Figure 7) and one resulted in a bony fracture upon seating. This was deemed to be a failure.

### 3.2. In Vivo Animal Placements

In total, 12 animal placements were attempted, and all achieved successful placement.

The trial-fit gauge was tested for fit along its block perimeter. Care was needed so as not to fracture the bony walls upon press-fit action and the removal of the trial-fit gauge. A need for surgical judgment was apparent if the trial-fit gauge was not fitting beyond approximately 75% of its depth.

The perimeter of the rectangular block osteotomy sites was adjusted accordingly, and the depth was increased using spade-shaped piezotomes to achieve full seating of the trial-fit gauge to approximately 75% of its depth.

At times, the placement stem of the trial-fit gauge was unscrewed with the block seated in position and the block was tapped into position mesially and distally (using the surface purchase points) and sometimes centrally through the central hole of the block, using a stainless-steel punch and a gentle, firm mallet tapping action, until the desired level of press-fit seating was achieved through the compression of the underlying trabeculae.

Upon the desired seating of the trial-fit gauge, the insertion stem was then re-attached with firm finger pressure and used to achieve gentle, firm mesiodistal rocking of the trial-fit gauge until it was removed. Care was taken not to cause buccolingual rocking, as this could cause a fracture of either of these bony plates.

The block implants were tapped into position as per the ex vivo placements.

Post seating, the insertion stem was re-attached and the block was checked for immobility with finger pressure. All placements achieved excellent primary stability.

**Figure 8:**
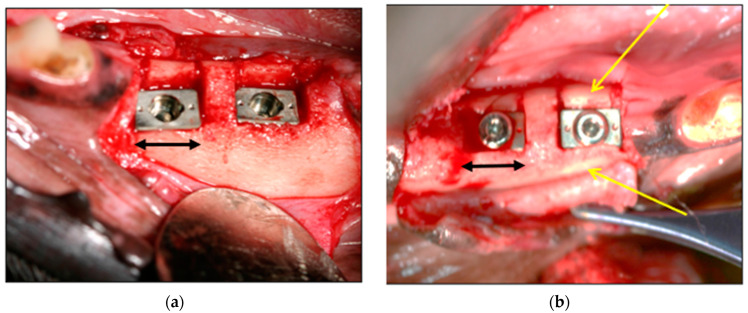
(**a**) Examples of Seated Implants. (**b**) Note the crestally overlapping bony buccal margins (yellow arrows). Black arrows represent relative 6 mm lengths.

Occasionally, the crestal bone was seen overlapping the seated implant (Figure 8). This was indicative of the elastic recoil (visco-elastic properties of bone) in the buccal and lingual bony margins upon final seating, which yielded excellent primary retention despite any over-extended saw cuts.

**Figure 9:**
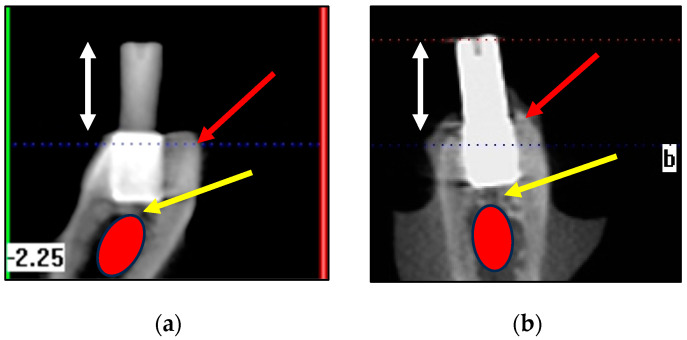
Examples of CBCT bucco-lingual views (**a**,**b**). CBCT confirmed good crestal bony coverage (red arrows). These bucco-lingual views highlighted the proximity (yellow arrows) of the “apical” base and the superior aspect of the inferior alveolar nerve canal (red ellipses). Given that the length of this implant is 5.25 mm, it emerged that a longer implant would be anatomically precluded. White arrows represent relative 6 mm lengths. Buccal orientation in these cross sections: “b”.

CBCT radiography confirmed the depths of the placements, the proximity of the flat RBI apical faces to the underlying mandibular canal and the presence of the buccal and lingual bony walls (Figure 9). The proximity to this IAN canal confirmed that there was very little vertical clearance for the placement of an implant any longer than the current one, of length 5.25 mm.

### 3.3. Human Placements

#### 3.3.1. Clinical Images

**Figure 10:**
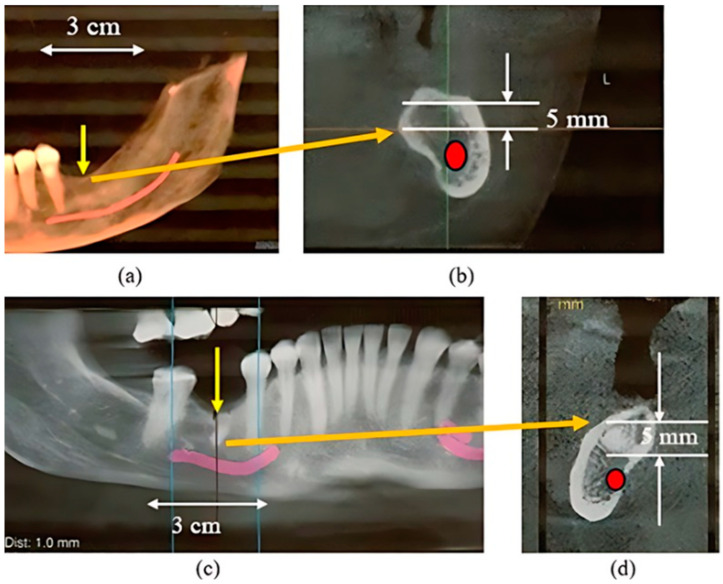
CBCT images Patients 1 (**a**,**b**), Patient 2 (**c**,**d**). (**a**,**c**): Extensive alveolar resorption at the planned sites (yellow arrows). (**b**,**d**): Cross-sectional views of the proposed positions of the planned RBIs where there was little crestal clearance (approximately 5 mm) above the inferior alveolar nerve (red).

**Figure 11:**
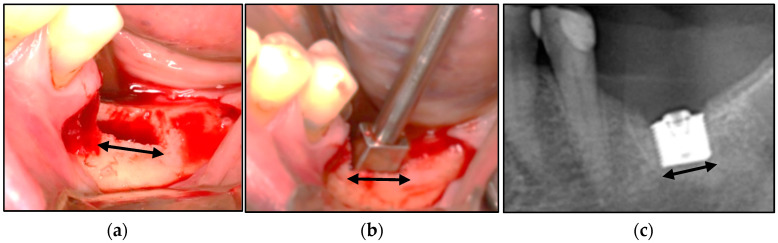
Patient 1. (**a**) The rectangular osteotomy. (**b**) The trial-fit gauge was tested repeatedly to achieve complete seating. (**c**) The RBI was inserted to full depth without bony fracture and achieved primary stability. This was visualised radiographically. Black arrows represent relative 6 mm lengths.

**Figure 12:**
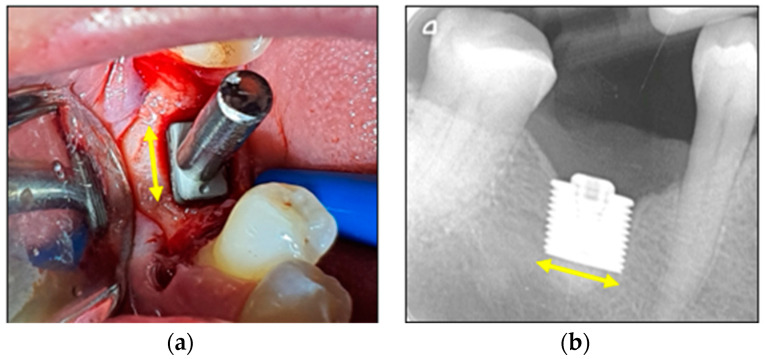
Patient 2. (**a**) Placement of the RBI into the osteotomy with a stem attached. Finger pressure on the stem confirmed excellent stability. (**b**) Radiographic image of the fully seated and well-stabilised RBI. Yellow arrows represent relative 6 mm lengths.

These cases involved the left mandibular resorbed alveolus: Patient 1 was a left-side placement, while Patient 2 was a right-side placement (Figure 10, Figure 11 and Figure 12).

#### 3.3.2. Statiscal Significance

The pooled total of the above-described placements yielded 16 successful placements and one unsuccessful placement (ex vivo animal: bone fracture). A binomial analysis based on the random outcome of either a successful or an unsuccessful surgical placement was applied to the pooled number of placements. The event of a random successful surgical outcome was rejected within the *p* < 0.01 confidence limit.

## 4. Discussion

The surgical placement of the RBI has a unique biomechanical perspective, which reflects its geometry. The main features of this are related to its flat-faced and cornered geometry, as well as its intended resorbed site osteotomy. These two aspects will now be considered.

It is clarified that the images in this discussion are not drawn to scale; they serve as explanatory schematics only.

### 4.1. RBI Geometry

The press-fit placement of the RBI is accomplished by tapping the block into position. These impact forces represent the placement loads and are initially sustained by peri-radicular bone as hoop stresses and are eventually distributed by the flat apical portion of the RBI. The flat faces act as potent distributors of the local stresses induced by the press-fit placement forces. Although the ideal press-fit placement force should be directed along the centroid of the block, given the limitations in surgical access in the posterior segments, in practice, some of the applied placement forces are obliquely oriented.

**Figure 13:**
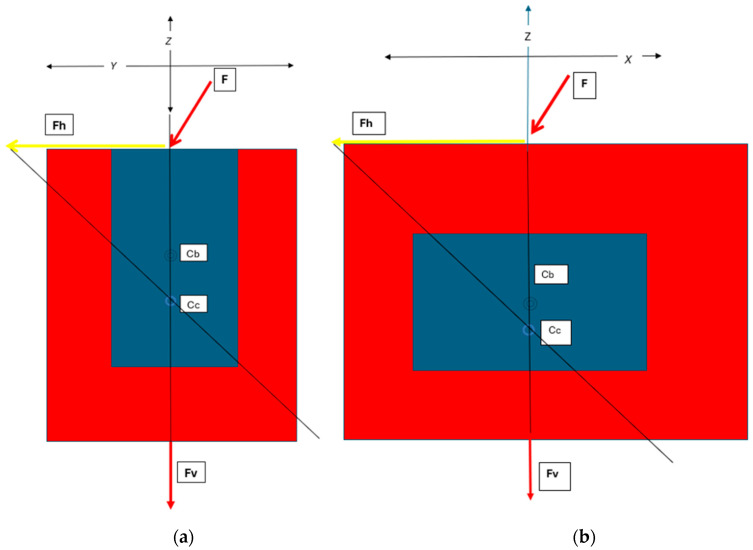
Schematic of the Horizontal (Fh) and Vertical (Fv) Components of the Applied Force (F):—as expressed through the centre of the RBI, which will also contain the centroid of the block-bone complex (Cc). The centroid will be apically positioned with respect to a normally free-bodied block centroid (Cb) due to the unison with the bone. These are depicted (**a**) from the buccolingual cross-sectional perspective and (**b**) from the mesiodistal cross-sectional perspective, as both aspects would have off-centred forces applied. X, Y and Z axes are depicted.

**Figure 14:**
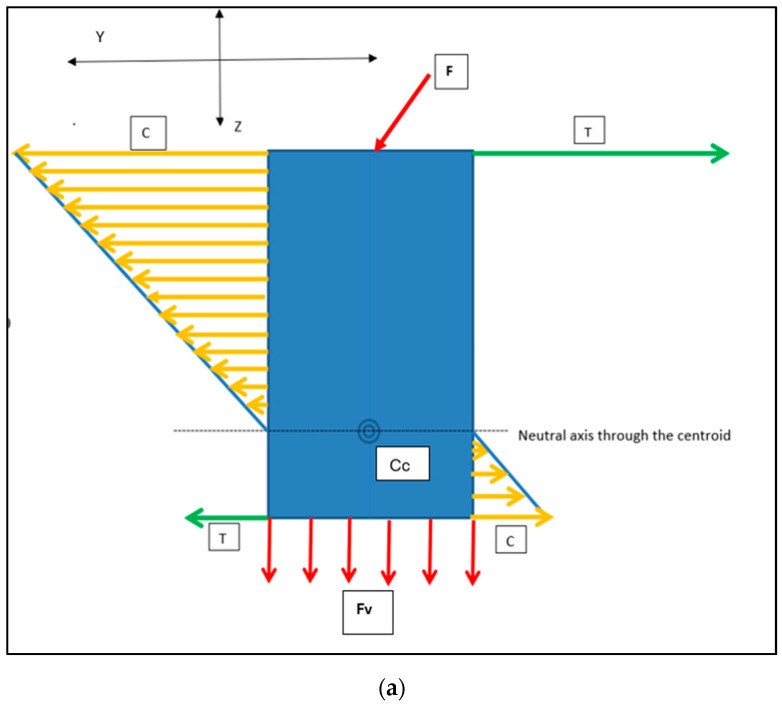

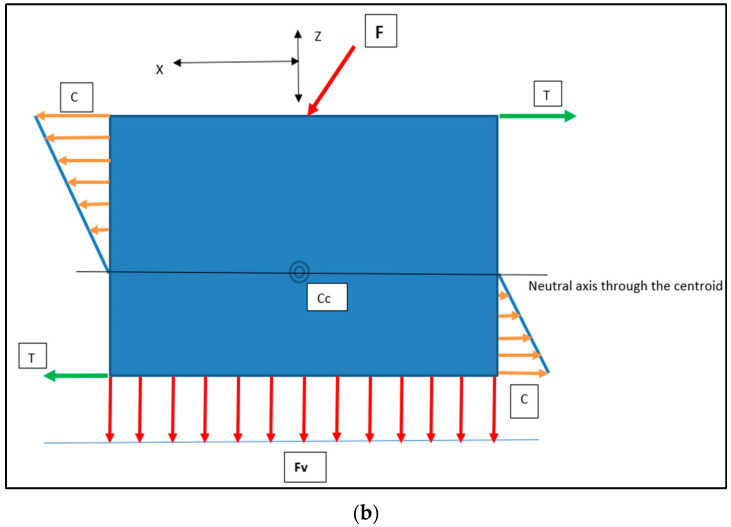
Load Intensity Schematic of the Horizontal (orange arrows) and Vertical Force (red arrows) Components: the crestal buccal (orange: compression [C]) and crestal lingual (green: tension [T]) equal and opposite reactions. Proceeding apically, these directions will be reversed below the centroid of the block–bone complex [Cc]. These are depicted (**a**) from the buccolingual cross-sectional perspective and (**b**) from the mesiodistal cross-sectional perspective. X, Y and Z axes are depicted.

The ISO 14801 [28] standard in testing implant biomechanical profiles has traditionally assumed a 30° off-axis orientation of applied loads as a worst-case scenario. This principle can also be applied to the insertion mechanics of the RBI as it is a press-fit action and designed for the posterior region where access is often limited and hence the insertion force will rarely be perfectly axially oriented. This will be relevant as an obliquely applied “vertical” (Z-axis) placement load with “horizontal” components in both the mesiodistal (X-axis) and the buccolingual (Y-axis) (Figure 13 and Figure 14).

**Figure 15:**
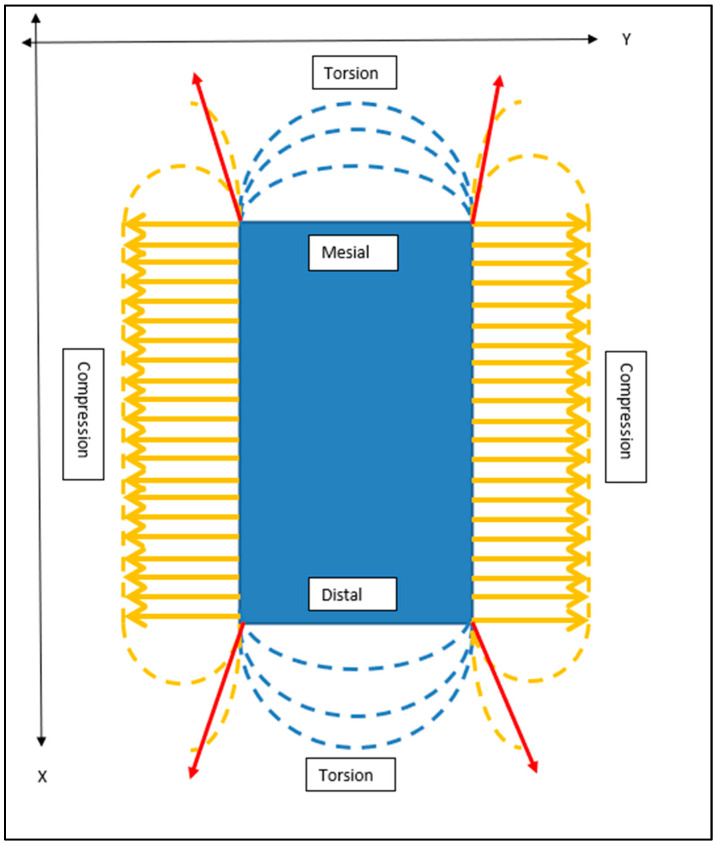
Schematic Outline of Crestal “Horizontal” Stress Patterns Generated Upon Complete RBI Placement. From the crestal perspective, the internal stresses generated on all four surfaces of the block resulting from the insertion load of the obliquely applied forces can be summarised as predominantly buccal and lingual plate compression (orange arrows). Stress concentrations will be maximal at the corners (red arrows) and approximal torsion. X and Y axes are depicted.

All loads will generate internal stresses, which will be buttressed in an equal and opposite manner by the surrounding bone, assuming negligible permanent displacement. Once fully seated and upon cessation of the placement force, residual implant-bone interface stresses will remain. This would be characterised by flat-faced perimeter compression and maximal stress concentrations at the corners. It is posited that the unison of these elements at the corners would yield torsional patterns at the mesial and distal extremities (Figure 15). This is supported by RBI fatigue analysis work performed in vitro [24], where RBIs were embedded in transparent epoxy resin and subjected to oblique cyclic loading up to 1000 N. Results showed unique mesial and distal-ended corner-to-corner semi-circular epoxy stress patterns suggestive of torsional stresses. Greater clarification of these observations requires further studies.

### 4.2. Cylindrical Implant Geometry

**Figure 16:**
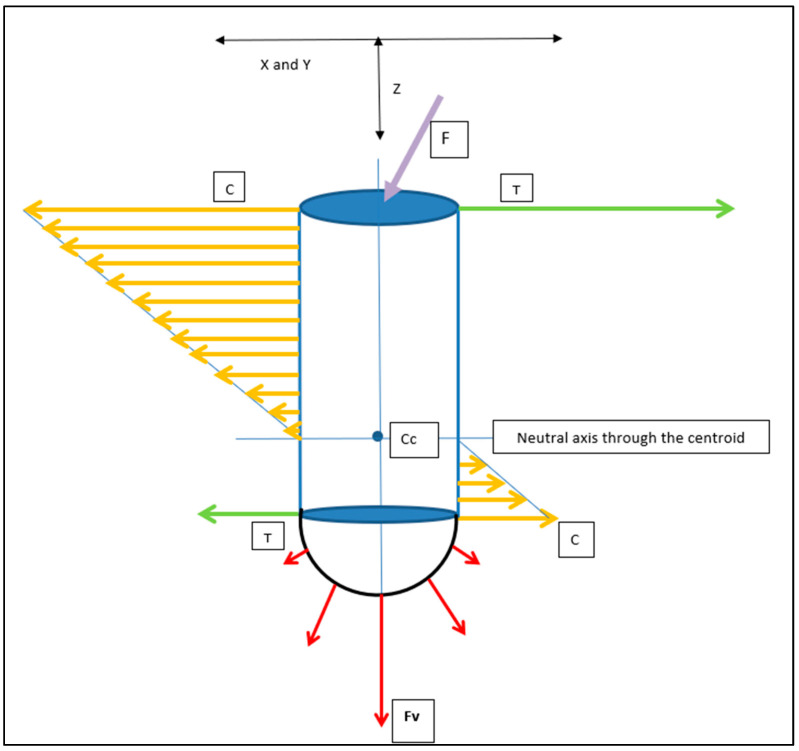
Cylindrical Implant Load Intensity Schematic. The applied load (F: Purple Arrow) generates Horizontal (compressive: orange arrows) and apical vertical (compressive: red arrows, (Fv)) force components. Compression (C) and Tension (T: green arrows) are in opposite directions and invert beyond the centroid of the implant–bone complex (Cc). X and Y axes would be symmetrical in the cylindrical geometry. X/Y and Z axes are depicted.

The cylindrical counterparts would have analogous but differing implant–bone interface stress patterns (Figure 16). Although the use of press-fit cylindrical implants is uncommon in today’s clinical landscape, this analysis remains useful as a general comparison in stress distribution between rectangular and cylindrical counterparts on oblique loading: both in press-fit placement and in functional load.

Both the horizontal and vertical force (Fv) distribution patterns will be unevenly distributed along the implant–bone interface.

**Figure 17:**
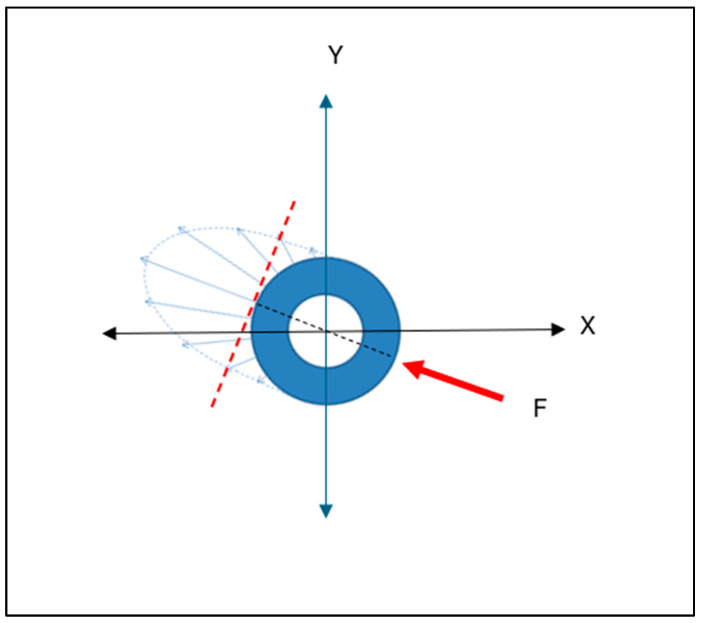
Cylindrical Implant Cross Sectional Schematic of the Horizontal Compressive Force Distribution. In any horizontal cross-section along the axial length of the cylindrical implant, the interfacial horizontal force distribution will be unevenly distributed. This distribution will be in an elliptical form (blue arrows), with a maximum at the radial position aligned with the direction of the applied force (F: red arrow), tangentially normal to the surface (hashed red line). X and Y axes are depicted.

The axis of maximal force concentration, resulting from the horizontal force component, will be normal to the tangential surface of maximal load. This is depicted in the cross-sectional (X and Y axes) view (Figure 17). This pattern is very different to that depicted above, for the flat-faced RBI.

### 4.3. Geometry Comparison

The critical difference from the cylindrical geometry (both for vertical and horizontal components) will be that at any given position along the bone-implant interface, the RBI flat faces will create an evenly distributed stress pattern. The cylindrical counterpart, however, will have a maximal stress value at any position tangential to the force interface, and this will dissipate exponentially (radially) away from this position.

**Figure 18:**
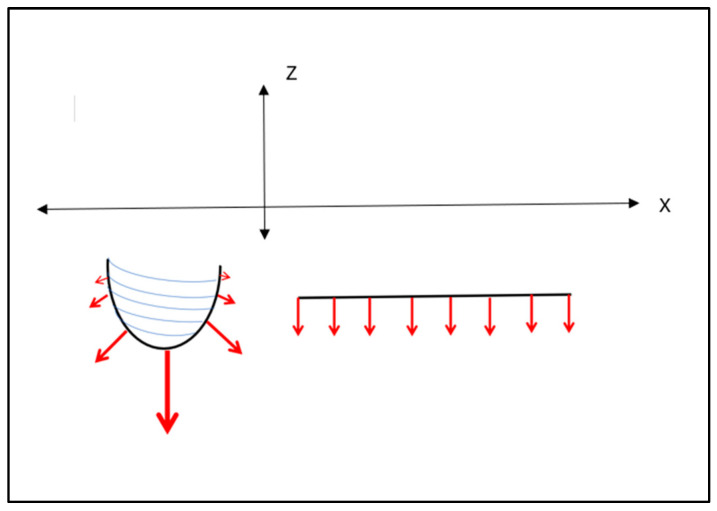
Apical Vertical Force Distribution (red arrows): Cylindrical (left) and Rectangular (right) Implants. It is seen that a cylindrical counterpart also has an analogous force intensity distribution, assuming the same uni-lateral and uni-directional applied force. The vertical component of the cylindrical implant will be concentrated along the apically directed axis and exhibit unequal distribution at the apex, approximating zero in the horizontal direction. The flat-faced counterpart will exhibit even apical distribution. X and Z axes are depicted.

Cehreli and co-workers [29] applied a uni-directional 150 N oblique force on various (cylindrical) implants mounted in epoxy resin and observed the internal stresses through isochromatic birefringence patterns generated by polarised light. Interfacial tensile and compressive stresses were clearly highlighted and concentrated at both ends. Although that study was not concerned with implant placements, its load analysis applies to all aspects of implant–bone interface load patterns. These authors showed that although the crestal stresses were dominant, the apical stresses were also highly significant. This is particularly pertinent given that the press-fit action of the RBI is predominantly in the axial direction, directed apically. The resultant apical stresses generated, however, will be different between the flat apical face of the RBI and the cylindrical screw shape (Figure 18). The vertical load (Z axis) will be evenly distributed across the “apical” face of the block.

### 4.4. Factors Influencing Press-Fit Placement

#### 4.4.1. The Press-Fit Action Limitations

The exact level of interference fit on press-fit placement remains ill-defined [19]. Abdul-Kadir et al. [30] have highlighted the Coulomb friction model of placement as a mechanical approximation of this process. The importance of this is the exponential nature of the frictional forces as placement proceeds; this has direct clinical ramifications affecting the accuracy of placements in deeper osteotomies and reducing the intra-operative recovery of inaccurate placement. Furthermore, bone deformation [17] and damage (compaction and abrasion) [19] are unavoidable consequences that require further clarification. These issues are magnified by the significant influence of individual bone quality, which renders the press-fit process highly dependent on operator judgement [30]. This has the potential to reduce the predictability of clinical outcomes.

Clinical judgement remains paramount generally in the assessment of primary retention at the time of placement [31]. This, however, is magnified in the press-fit action since reverse torque is not applicable, and pull-out actions risk jeopardising the entire procedure.

#### 4.4.2. Press-Fit Stress Patterns and the RBI

Frisardi et al. [20] conducted finite element analysis of press-fit implant placements in under-prepared cylindrical osteotomies using the isotropic characterisation of cortical and cancellous bone generated from micro-computerised tomography (μ-CT) voxels. They found that radial under-preparation of approximately 0.35mm generated stresses of 7.75 GPa and corresponding strains (ε = 0.51) that were mainly focused at the crestal cortical bone (coefficient of friction μ = 0.2). Crucially, these authors clarify that the Von Mises stress distributions, where the interference fit is maximised, show that the press-fit phenomenon is almost completely supported by the cortical structure, even though the implant is in contact with both the cortical and the trabecular bone. The stresses on the trabecular bone were minimal compared to those characterising the stresses on the cortical bone. This stress pattern was diminished only when the osteotomy was enlarged to minimise the interference fit. Clinically, this would mitigate primary retention.

It emerges that at the crestal position, the horizontal elements (as per Cehreli et al. [29]) are critical, and this underpins the mechanism of lateral (horizontal) bone fracture in the under-prepared site.

The even-faced load distribution for the RBI, however, is also counteracted by the stress concentrations at its corners. This unfavourable aspect of the RBI is a trade-off with enhanced primary retention generated by the corners of the flat faces once the RBI has achieved full seating, that is, a “locking” of the RBI into the osteotomy site facilitated by the cornered geometry [25].

The importance of the above-discussed stress patterns is further magnified given that firstly, the osteotomy sites chosen are within the resorbed ridge. That is, the resultant buccal and lingual walls are thin and more prone to fracture, as well as being more cortical in nature. Secondly, the RBI placement is a press-fit action and hence expands the investing bone, generating peri-implant compressive forces. A cylindrical counterpart would firstly, simply have radial stress patterns and without concentrations at the corners. Secondly, these would be further minimised by the radial drilling action of the site preparation.

Of primary relevance also is the density of the bone itself and the effect this has on the buccal and lingual bony torsional strength. It follows that the extent of expansion must be within the visco-elastic capacity of the investing bone. This, in turn, is related to its mineral density, which has been quantified at an ideal range of 1.1–1.2 g/cm^3^ [32], its maximum cortical thickness of approximately 2 mm [32] and its overall longitudinal and transverse modulus of elasticity (approximately 10–30 GPa) [33].

Notwithstanding the importance of these factors, they remain ill-defined with respect to the clinical insertion of endosseous implants generally, and their applied roles require clarification. This is beyond the scope of the present study.

This discussion further highlights the pivotal nature of the mechanical osteotomy preparation itself. The osteotomy must be large enough to allow complete seating without generating buccolingual stresses beyond the osseous elastic capacity yet small enough to allow primary retention. Furthermore, the nature of the bone itself will vary depending on the patient, and the above biomechanical concepts cannot be defined in exact terms.

Press-fit implant biomechanics are also influenced by the surface roughness of the titanium. There is a corresponding direct relationship between this and the bone implant coefficient of friction at the moment of interference fit. Although this is beyond the scope of this discussion, it is pertinent that orthopaedic research has highlighted that beyond the initial placement, excessive roughness of the fixture surface may play a negative role in the achievement of an interference fit that resists pull-out force at the time of placement [17]. The combined effects of the parameters of surface roughness, geometrical shape and bone nature on press-fit biomechanics bring to the fore the need for studies that can simulate the intra-osseous stresses generated and predict strains prior to surgical placement. There are few finite element analysis (FEA) studies on press-fit dental implants to date, and these have only been performed on cylindrical implants [20]. The present report highlights the need for FEA of the cortical bone stresses emanating from the RBI press-fit process.

### 4.5. Piezosurgery

The mechanical role of the piezo surgical instrumentation is also important. Firstly, it facilitates the saw-like cutting of bone through preferential specific vibrational frequency, and this allows both linear cuttings as well as preservation of any inadvertent soft tissue impingement. This is further magnified by the customised piezo tools which have cutting teeth which are both 0.5 mm in height and emanate from a flat platform. The result is a self-limiting mechanical cutting action that is crucial at the depth of the osteotomy as it approaches the superior border of the mandibular canal. Hence, the slow, repeated use of this tool at full depth acts as a biomechanical safeguard against inferior alveolar nerve damage.

### 4.6. Future Research

#### 4.6.1. Graeter Sample Sized Studies

Further studies are needed with greater numbers of surgical samples that will clarify the exact role of the osteotomy size and the stresses and strains generated in the investing bone upon implant seating. This would ideally be performed in natural bone, and to this effect, animal studies with cortical strain gauge measurements at the time of seating are needed. Extrapolation to human placements requires larger sample sizes based on multi-centre surgery, as well as a detailed pre-operative bone density assessment.

#### 4.6.2. Primary Stability

A quantitative assessment of primary stability is also needed, and to this effect, the use of resonance frequency analysis (RFA) [34,35] upon placement may serve as a useful tool. To date, however, the use of RFA at the time of placement as a prognostic indicator of later implant success remains contentious [36].

#### 4.6.3. Titanium Surface Modifications

The limitations discussed above concerning bone quality and factors that affect the press-fit osseointegration are particularly pertinent for the RBI, which is classified as a “short” implant [37]. It follows that surface modifications that either bio-actively enhance osseointegration [38] or preserve it by protecting it from bacterial invasion [39] will ultimately increase bone-to-implant contact and the biomechanical outcome of the placement. Future studies are needed to be designed to embody such surface modifications and assess their enhanced outcome of the press-fit RBI placement.

#### 4.6.4. Forces Distributions under Differing Conditions

This is the first attempt relating to press-fit dental implants to schematically define biomechanical force gradients, and as such is a simplistic representation. Further studies are needed that present a greater analysis of peri-implant press-fit force gradients both quantitatively and under varied clinical conditions (e.g., compromised bone density and osteoporotic bone). Ideally, these simulations should pre-exist and have the capacity to determine the limits of the RBI application and thus minimise the real risk of bone fractures. Although pull-out tests have been presented [25], future pull-out simulations are also needed to reflect these varied clinical conditions. Such pull-out studies should also embody analogous force distribution analysis, which would complement the press-fit action.

## 5. Conclusions

The biomechanical considerations of the experimental surgical protocol verified the following concluding statements.

The use of the piezo-surgical saw and customised rectangular-shaped piezotomes were capable of producing a rectangular-shaped trough osteotomy that matches the RBI. Although the piezo-surgical action acted to cut bone, it did not damage adjacent soft tissues and acted to facilitate the efficient creation of the rectangular osteotomy.

The use of a trial-fit gauge with slightly smaller dimensions than the RBI was helpful in acting as a template for the osteotomy outline and dimensions such that the trial-fit gauge would be able to fit passively into the surgical defect to approximately 75% of its depth. Underpreparation of the osteotomy may result in a fracture of the buccal and/or lingual bone walls upon the press-fit action of the RBI.

The final press-fit of the RBI with correctly sized osteotomes and mallet actions resulted in the complete seating of the RBI.

Although not quantified, it is posited that the flat RBI faces act to limit stress concentration, and this is especially pertinent in the apical segment.

The cornered surfaces of the RBI may, however, act as both stress concentrators and primary stability enhancement. The action of these requires further investigation. The overall effect of the cornered flat surfaces combined with the under-preparation of the osteotomy yielded excellent primary stability. Notwithstanding this, the visco-elastic nature of the buccal and lingual bony walls themselves permitted the attainment of primary stability of the press-fit block, as evidenced by applied manual force.

More studies are needed to quantify the above factors, including bone density, strain gauge and possibly resonance frequency analyses.

## Data Availability

All data created or analyzed are contained within this study.
